# Evidence of neuroinflammation and immunotherapy responsiveness in individuals with down syndrome regression disorder

**DOI:** 10.1186/s11689-022-09446-w

**Published:** 2022-06-03

**Authors:** Jonathan D. Santoro, Rebecca Partridge, Runi Tanna, Dania Pagarkar, Mellad Khoshnood, Mustafa Rehmani, Ryan M. Kammeyer, Grace Y. Gombolay, Kristen Fisher, Allison Conravey, Jane El-Dahr, Alison L. Christy, Lina Patel, Melanie A. Manning, Heather Van Mater, Michael S. Rafii, Eileen A. Quinn

**Affiliations:** 1 Division of Neurology, Department of Pediatrics, Children’s Hospital Los Angeles, 4650 Sunset Blvd, MS82, Los Angeles, CA 90027 USA; 2 Department of Neurology, Keck School of Medicine at the University of Southern California, Los Angeles, CA USA; 3 Down Syndrome Program at Virginia Mason, Seattle, WA USA; 4 Keck School of Medicine at the University of Southern California, Los Angeles, USA; 5 Department of Psychiatry, Keck School of Medicine at the University of Southern California, Los Angeles, CA USA; 6 Department of Neurology, University of Colorado, Aurora, CO USA; 7 Department of Neurology, Children’s Healthcare of Atlanta, Atlanta, GA USA; 8 Emory University School of Medicine, Atlanta, GA USA; 9 Division of Neurology, Cincinnati Children’s Hospital, Cincinnati, OH USA; 10 Ochsner Health, Department of Pediatrics, New Orleans, LA USA; 11 Section of Pediatric Allergy, Department of Pediatrics, Tulane University School of Medicine, New Orleans, LA USA; 12 Providence Health, Portland, OR USA; 13 Department of Psychiatry, University of Colorado Anschutz Medical Campus, Aurora, CO USA; 14 Division of Medical Genetics, Stanford University School of Medicine, Palo Alto, CA USA; 15 Division of Rheumatology, Department of Pediatrics, Duke University, Durham, NC USA; 16 Alzheimer’s Therapeutic Research Institute (ATRI), Keck School of Medicine at the University of Southern California, San Diego, CA USA; 17 Department of Pediatrics, University of Toledo College of Medicine and Life Science, Toledo, OH USA

**Keywords:** Down syndrome, Encephalopathy, Immunotherapy, Cerebrospinal fluid, Regression

## Abstract

**Background:**

Down syndrome regression disorder is a symptom cluster consisting of neuropsychiatric regression without cause. This study evaluated the incidence of neurodiagnostic abnormalities in individuals with Down syndrome regression disorder and determined if abnormalities are indicative of responses to therapeutic intervention.

**Methods:**

A retrospective, multi-center, case-control study was performed. Patients were required to have subacute onset and the presence of four of five symptom groups present (cognitive decline, expressive language, sleep derangement, loss of ability to perform activities of daily living, and/or a new movement disorder) and no other explanation for symptoms.

**Results:**

Individuals with Down syndrome regression disorder were comparable to a cohort of individuals with only Down syndrome although had higher rates of autoimmune disease (*p* = 0.02, 95%CI 1.04–1.75). Neurodiagnostic abnormalities were found on EEG (*n* = 19, 26%), neuroimaging (*n* = 16, 22%), and CSF (*n* = 9, 17%). Pleocytosis was appreciated in five cases, elevated total protein in nine, elevated IgG index in seven, and oligoclonal bands in two. Testing within 2 years of symptom onset was more likely to have neurodiagnostic abnormalities (*p* = 0.01, 95%CI 1.64–37.06). In individuals with neurodiagnostic abnormalities, immunotherapy was nearly four times more likely to have a therapeutic effect than in those without neurodiagnostic abnormalities (OR 4.11, 95%CI 1.88–9.02). In those with normal neurodiagnostic studies (*n* = 43), IVIg was effective in 14 of 17 (82%) patients as well although other immunotherapies were uniformly ineffective.

**Conclusions:**

This study reports the novel presence of neurodiagnostic testing abnormalities in individuals with Down syndrome regression disorder, providing credence to this symptom cluster potentially being of neurologic and/or neuroimmunologic etiology.

**Supplementary Information:**

The online version contains supplementary material available at 10.1186/s11689-022-09446-w.

## Background

Down syndrome (DS) is the most common cause of intellectual disability worldwide and occurs in 1 in 800 live births [1]. Neurologic and psychiatric diseases in this population are well established, although over the last decade an increasing frequency of reports of the onset of subacute developmental regression of unclear etiology in individuals considered too young to develop Alzheimer’s disease has been reported [2, 3]. This condition has been referred to as Down syndrome disintegrative disorder (DSDD) or Down syndrome regression disorder (DSRD) and “unexplained regression in Down syndrome” (URDS) [4] and has primarily been reported in young persons with DS between ages 10 and 30 years. Clinical phenomenology consists of a subacute loss of previously acquired developmental skills in the areas of language, communication, cognition, executive function, behavioral, and adaptive skills [2–5]. Other symptoms can include psychiatric manifestations, motor symptoms (stereotypies, extrapyramidal), catatonia, and rapid onset insomnia [2, 4–7]. DSRD can be severe and significantly impact both the quality of life and autonomy of persons with DS and their families.

Neither epidemiologic studies nor concentrated searches for disease biomarkers have been performed in persons with DSRD, making the etiology of this symptom cluster difficult to ascertain. Neurologic, psychiatric, genetic, and neuroimmunologic etiologies have been proposed [8] although many of these hypotheses are largely based on the etiology of regression in other forms of neurodevelopmental disorders and intellectual disability [9–14]. A lack of consistent criteria or diagnostic approach to DSRD exists at this time, yielding heterogeneity of diagnostic and neurodiagnostic investigations which has limited generalizability of published reports.

This study sought to investigate the potential role of neurologic and neuroimmunologic dysfunction in persons with DSRD by determining the prevalence of neurodiagnostic abnormalities in persons with DSRD and whether the presence of these findings dictates response to particular therapeutic interventions.

## Methods

### Patient population

Patients were retrospectively identified following IRB approval through an institution-based audit of in-person evaluations and telemedicine-based consultations for persons with DS and neurocognitive regression from July 1, 2019, to October 1, 2021. Remote sites (Additional file 1: Appendix 1) contributed cases that were similarly identified through the course of practice as there is no unique ICD9 or ICD10 code for DSRD.

### Inclusion criteria

All patients were required to have acute or subacute onset of symptoms (defined as 0–12 weeks), a genetically confirmed diagnosis of DS, and an EEG, MRI, and lumbar puncture (LP) performed while symptomatic. In addition, as there are no definitive diagnostic criteria for DSRD, patients required at least four of five total clinical features: (1) cognitive decline (defined as any new deficit in memory, processing speed, attention, or awareness), (2) decreased expressive language or mutism, (3) new-onset insomnia or hypersomnia, (4) loss of ability to perform activities of daily living, and/or (5) catatonia or a movement disorder (excluding tics). An a priori benchmark of decline of at least 50% of prior functional status reported by parents or caregivers was required for inclusion.

### Exclusion criteria

Patients with a history of any prenatal neurologic insult, prematurity (defined as birth < 36 weeks gestation), known/established structural malformation of the brain, history of leukemia/lymphoma, receipt of chemotherapy and/or ionizing radiation, history of any neurologic disorder (e.g., infantile spasms, epileptic encephalopathy, stroke), history of complex congenital heart disease requiring surgical intervention (with the exception of ventricular septal defect, atrial septal defect, or patent foramen ovale), or untreated thyroid, cardiac, or obstructive sleep apnea (OSA) were excluded. Finally, cases with insufficient data (see neurodiagnostic workup) were also excluded.

### Demographic comparison

Individuals with DSRD were compared to individuals with only DS. This information was obtained from a multi-institutional retrospective database of persons with DS, and the same exclusion criteria were applied. Inclusion in the comparator group only required a prior diagnosis of DS with enrollment designed to longitudinally follow health outcomes in individuals with DS. Individuals with DSRD were not pooled into the DS comparator data and were manually extracted by medical record number identification. As nearly all patients referred for EEG or lumbar puncture met exclusionary criteria, no comparison could be made on these diagnostic tests. However, neuroimaging data was collected in patients in the comparator group when no exclusionary criteria were met. All patients required prior imaging on a 3-T MRI scanner.

### Clinical assessments and data extraction

All patients were evaluated by a board-certified pediatrician, geneticist, and/or neurologist. The primary evaluator documented the presence or absence of each symptomatic diagnostic criterion referenced, and assessment of each was made by a combined approach of reviewing clinical history (provided by family or caregiver), home videos of prior function, review of school-based forms, reports of other evaluating physicians (e.g., primary care, emergency room), and physical and neurologic examination. Demographic and clinical data were extracted from these encounters and reviewed manually by the authors in a blinded fashion (JDS, MR, RT) for extraction of established phenotypes in DSRD [4]. All data was systematically reported, and when not documented in the medical record, a query was made to the evaluating clinician for clarification by the first author (JDS). Symptoms were clustered into eight groups (Additional file 2: Appendix 2). The inclusion criteria used in this study included symptoms from this larger grouping although not all symptoms were used in the inclusion criteria as both author group and external expert review (JDS, GYG, KF, ALC, HM MSR, EAQ, please also see acknowledgments) determined that these symptom clusters were best utilized for descriptive, as opposed to diagnostic, purposes. The presence of a symptom cluster in an individual was defined by having > 50% of the symptoms within any grouping. Each case was reviewed by two authors with a third utilized only when there was discrepant data extracted from a case. To avoid bias in the interpretation of therapeutic responses, a review of data on therapeutic responses was made without knowledge of the diagnostic abnormalities or prior therapies trialed.

### Neurodiagnostic workup

As this study was retrospective in nature, not all neurodiagnostic workups were executed at the same time points or in the same order. Definitions of EEG, MRI, and LP abnormalities are listed in Additional file 3: Appendix 3.

### Therapeutic responses

Quantification of clinical improvement with specific therapeutic interventions was subjectively assessed from documentation by the research team. Clinical response was assessed as any of the following: (1) subjective report by patient, parent, or guardian; (2) resolution of lab or neurodiagnostic study abnormality (e.g., normalization of EEG); (3) discontinuation of medications when clinical improvement was cited as a cause; or (4) improvements on physical examination or neurologic examination (e.g., expressive language documented in a patient with prior mutism). All evaluating clinicians provided an interpretation of response based on a percentage (e.g., 50% improved) between visits and since the initial evaluation. When multiple scores were provided (e.g., 50% improvement in expressive language, 30% improvement in gait), the scores were averaged for the visit. Improvement based on the resolution of lab or neurodiagnostic abnormalities or discontinuation of medication were considered binary (yes = improved, no = not improved). As not all patients had repeat neurodiagnostic testing, analysis was only provided for those who had repeat studies performed. Discontinuation of medications was assessed through the changes in the medication administration record (MAR) and clinical reports. Only medications that were originally prescribed for the treatment of DSRD were assessed for discontinuation (e.g., antibiotics prescribed for sinusitis were not included). Finally, physical and neurologic examination changes were assessed through a review of medical records between encounters by a clinician (JDS, MR, MK). Non-validated metrics for the assessment of DSRD (e.g., Bush-Francis Scale) were not considered with regard to the clinical assessment for physical examination changes. Therapeutic responses were reviewed by two authors with a third utilized only when there was discrepant agreement on a case. Individuals treated with more than one therapy required at least 4 weeks of treatment without multiple therapeutic changes to be classified as effective/ineffective.

### Statistical analysis

Descriptive statistics were produced for demographic and clinical presentations. Cohen’s kappa was used to assess inter-rater reliability for the inclusion/exclusion of cases and in clinical data extraction. Interquartile ranges were calculated for continuous variables. Chi-squared analysis was performed for evaluating the differences between the sub-groups. Odds ratios with corresponding 95% confidence intervals were used to calculate the likelihood of therapeutic benefit between individuals with neurodiagnostic abnormalities and those without. A *p* nominal value of < 0.05 was considered statistically significant for each statistical test.

## Results

In total, 97 cases were identified for the potential review of which 74% (72/92) met the criteria for inclusion. The most common reasons for exclusion were incomplete data (68%, *N* = 17/25), prior diagnosis of a neurologic disorder (28%, *N* = 7/25), and prior receipt of chemotherapy (8%, *N* = 2/25). The median number of physician evaluations prior to the diagnosis of DSRD was 4, including primary care (IQR 3–6). Clinical data extracted by the authors yielded a Cohen’s kappa coefficient of 0.73 (91% agreement).

**Fig. 1:**
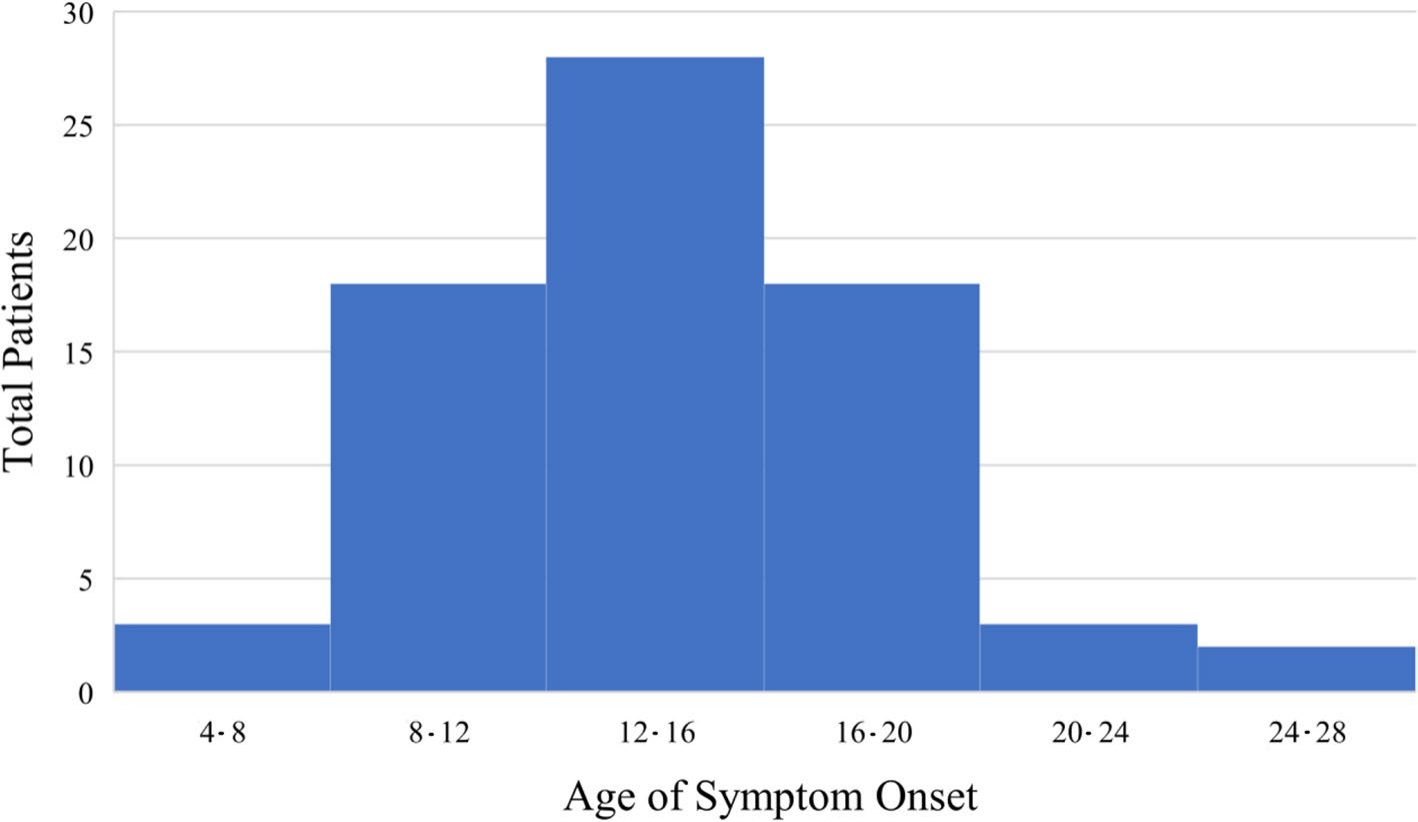
Histogram of symptom onset by age

Demographic data are presented in Table 1. The median age of onset was 14 years (IQR 12–17) with the median age at diagnosis being 19 years (IQR 13–27) (Fig. 1). The majority of this cohort was Caucasian (76%, *N* = 55/72) with Hispanic ethnicity (71%, *N* = 51/72), the latter being at a higher rate than our control population (*X*^2^(1, *N* = 1289) = 50.5, *p* < 0.001, 95%CI 1.96–2.76). Individuals with DSRD were more likely to have a history of autoimmune disease (*p* = 0.02, 95%CI 1.04–1.75) and thyroid disease (*p* < 0.001, 95%CI 1.64–3.27) compared to individuals with DS without regression.

**Table 1:** Demographics and clinical features

	DSRD (***n***= 72)	DS (***n***= 1217)	***p*** value	95%CI
Sex				
*Male* *Female*	35 (49%) 37 (51%)	578 (47.5%) 638 (52.5%)	0.85	0.80–1.31
Race				
*Caucasian* *Asian* *Black*	55 (76%) 10 (14%) 7 (10%)	1058 (87%) 109 (9%) 49 (4%)	0.06 0.15 0.22	0.77–1.01 0.85–2.83 0.13–5.14
Hispanic ethnicity	51 (71%)	370 (35%)	**< 0.001**	**1.96**–**2.76**
Medical history				
*Obstructive sleep apnea (n= 70)* *Congenital heart disease* *Autoimmune disease (any)* *Thyroid disease*	35 (50%) 38 (53%) 33 (46%) 25 (34%)	523 (43%) 548 (45%) 426 (35%) 182 (15%)	0.22 0.12 **0.02** **< 0.001**	0.91–1.48 0.96–1.51 **1.04**–**1.75** **1.64**–**3.27**
Age at onset (median, IQR)	14 (12–17)	11 (7–15)^a^	**< 0.001**	**1.05**–**3.55**
Preceding trigger *Infection*^*b*^ *Change in school/work/home environment* *Loss of family/caregiver/friend* *Change in residence* *Abuse* *Death in immediate social network* *Medical change*	37 (51%) 16 (43%) 10 (27%) 4 (10%) 2 (5%) 2 (5%) 2 (5%) 1 (3%)			
Months to symptom peak (median, IQR)	3 (1–6)			
Clinical symptoms				
*Social withdrawal* *Loss of acquired skills* *New autistic features* *Diminished eye contact* *Apathy (n = 70)* *Impaired attention* *Anxiety* *Mutism/expressive language regression* *Insomnia* *Anorexia* *Whispered speech* *Confusion/disorganization* *Memory impairment* *Catatonia* *Freezing/bradykinesia* *Abulia/avolition (n = 71)* *Emotional lability (n = 61)* *Stereotypy* *Urinary retention* *Incontinence (urine/feces)* *Inappropriate/mirthless laughter (n = 65)* *Circadian rhythm alteration* *New obsessive-compulsive tendencies* *Tics (n = 69)* *Aggression/agitation (n = 71)* *Hyperactivity* *Autonomic dysfunction (n = 58)* *Persistent focal neurologic deficits* *Seizure (n = 71)* *Transient neurologic deficits/TIA*	72 (100%) 72 (100%) 72 (100%) 69 (96%) 66 (94%) 64 (89%) 62 (86%) 59 (82%) 58 (81%) 56 (78%) 56 (78%) 55 (76%) 55 (76%) 55 (76%) 52 (72%) 46 (65%) 37 (61%) 38 (53%) 38 (53%) 36 (50%) 34 (52%) 26 (36%) 24 (33%) 19 (28%) 19 (27%) 11 (15%) 8 (13%) 3 (4%) 1 (1%) 1 (1%)			

*CI* confidence interval, *DS* Down syndrome, *DSRD* Down syndrome regression disorder, *IQR* interquartile range, *TIA* transient ischemic attack

^a^Age at data collection

^b^Any non-life-threatening bacterial or viral infection (e.g., upper respiratory tract infection, urinary tract infection, or pneumonia)

Clinical phenotypes are presented in Table 1. A potential preceding trigger was observed in 51% (*N* = 37/72) with recent infection reported in 43% of these (*N* = 16/37). The median time to peak symptoms was 3 months although this was heterogeneous with an IQR of 1–6.

**Fig. 2:**
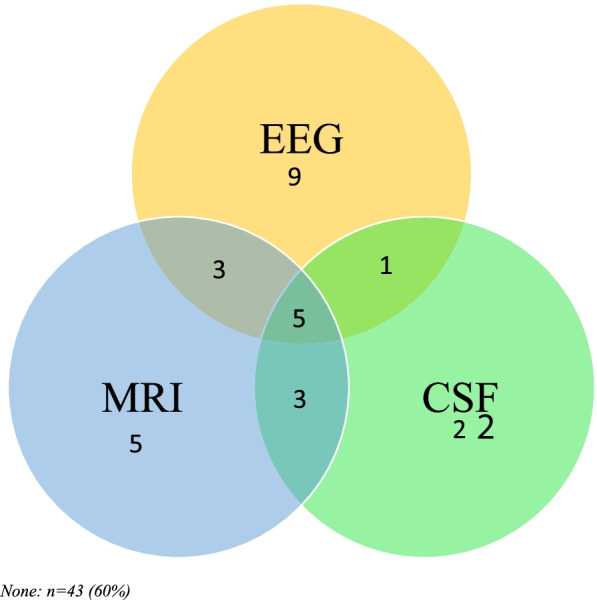
Number of patients with neurodiagnostic study abnormalities (*n*). None: *n* = 43 (60%)

Serum and neurodiagnostic data are presented in Table 2, and an overlap of neurodiagnostic study abnormalities is presented in Fig. 2. Serum analysis revealed thyroid peroxidase (TPO) and thyroglobulin antibodies were present in 37% (*N* = 25/72) and 30% (*N* = 20/72) of individuals, respectively, although only TPO antibodies were significantly greater than controls (*p* = 0.02, 95%CI 1.06–2.16). Vitamin D 25-OH levels were also significantly lower in individuals in the DSRD cohort compared to individuals with DS only (*n* = 384) (*p* < 0.001, 95%CI 10.57–16.9). Analysis of cytokine profiling revealed abnormalities in 40% (*N* = 20/50) of individuals tested with elevations in soluble IL-2 receptor (62%, *N*= 13/20) and IL-10 (24%, *N* = 5/20) most frequently observed. Compared to a limited cohort of 24 individuals with DS who had cytokine testing, the presence of any abnormality was significantly elevated in persons with DSRD (*p* = 0.02, 95%CI 1.23–17.74).

**Table 2:** Serum and neurodiagnostic studies

Study	DSRD results abnormal (***n***, %)	DS results abnormal (n/N, %)	***p*** value	95%CI
*Serum analysis*				
ANA	9 (13%)	21/422 (5%)	**0.01**	**1.20**–**5.26**
Anti-DNAseB	0 (0%)	1/125 (1%)	0.70	0.11–27.95
ASO (*n* = 58)	0 (0%)	6/204 (3%)	0.47	0.05–3.87
B12 level	7 (12%)	64/1125 (6%)	0.17	0.79–4.05
Celiac panel	2 (3%)	24/506 (5%)	0.46	0.13–2.48
Complete metabolic profile	5 (7%)	88/1256 (7%)	0.98	0.39–2.52
CRP (*n* = 62)	0 (0%)	15/433 (3%)	0.36	0.05–2.98
dsDNA (*n* = 61)	6 (10%)	3/108 (3%)	0.11	0.77–13.12
ESR (*n* = 66)	0 (0%)	16/612 (3%)	0.53	0.07–3.96
Infectious screen^a^	12 (18%)	n/a	n/a	n/a
Neopterin (*n* = 42)	0 (0%)	0/12 (0%)	n/a	n/a
Methylmalonic acid (*n* = 61)	9 (21%)	14/203 (7%)	0.15	0.79–4.67
Neurometabolic studies* (*n* = 32)	1 (2%)	3/188 (2%)	0.90	0.09–8.49
Thyroid dysfunction (untreated)	2 (4%)	160/842 (19%)	**0.01**	**0.04**–**0.58**
TPO antibodies (*n* = 43)	25 (37%)	110/478 (23%)	**0.02**	**1.06**–**2.16**
Thyroglobulin antibodies (*n* = 42)	20 (30%)	107/465 (23%)	0.37	0.80–1.82
Vitamin D (median, IQR)	26.5 (15–34)	39 (32–47)	**< 0.001**	**10.57**–**16.9**
Cytokine analysis (*n* = 50) *TNF-alpha* *IL-2* *sIL-2 receptor* *IL12* *Interferon gamma* *IL-4* *IL-5* *IL-10* *IL-13* *IL-17* *IL-1beta* *IL-6* *IL-8*	20 (40%) 1 (5%) 0 (0%) 13 (62%) 0 (0%) 1 (5%) 0 (0%) 0 (0%) 5 (24%) 0 (0%) 0 (0%) 0 (0%) 1 (5%) 0 (0%)	3/24 (13%) 0 (0%) 0 (0%) 0 (0%) 0 (0%) 1 (4%) 0 (0%) 0 (0%) 2 (8%) 0 (0%) 0 (0%) 0 (0%) 0 (0%) 0 (0%)	**0.02**	**1.23**–**17.74**
*Neurodiagnostic studies (DSRD only)*				
EEG abnormalities		19 (26%)		
*Epileptiform discharges in frontal/temporal lobes*		11 (58%)		
*Diffuse slowing (non-focal)*		6 (32%)		
*Focal slowing*		2 (11%)		
Neuroimaging abnormalities		16 (22%)		
*Punctuate T2 hyperintensities (gray/white junction)*		13 (81%)		
*Basal ganglia calcifications*		2 (13%)		
*T2 signal prolongation in the temporal lobes*		1 (9%)		
*Both T2 hyperintensities and calcifications*		1 (9%)		
*Contrast enhancing lesions*		0 (0%)		
CSF abnormalities *(n, %, median if abnormal)*		9 (17%)		
*WBC*		5 (7%, 6)		
*RBC*		0		
*Glucose*		0		
*Protein*		9 (13%, 68)		
*Oligoclonal bands (n = 60)*		2 (3%, 2)		
*IgG index (n = 60)*		7 (10%, 0.70)		
*Mayo autoimmune encephalitis panel (n = 59)*		0		
*Neopterin (n = 43)*		6 (8%, 45)		
Any abnormal neurodiagnostic study?		29 (40%)		
*> 2 abnormal studies*		12 (17%)		

*CI* confidence interval

*Neurometabolic studies included lactate, pyruvate, serum amino acids, urine organic acids, free and total carnitine, and acylcarnitine profiling

**Fig. 3:**
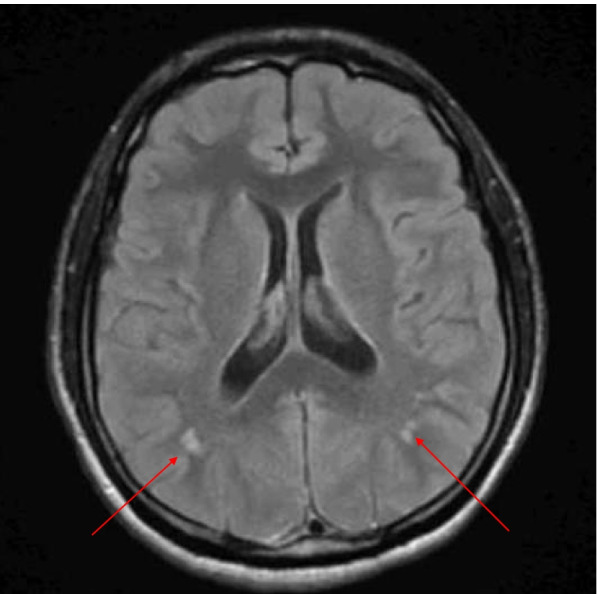
Axial T2 FLAIR sequence demonstrating T2 signal prolongation along the gray, white junction bilaterally

**Fig. 4:**
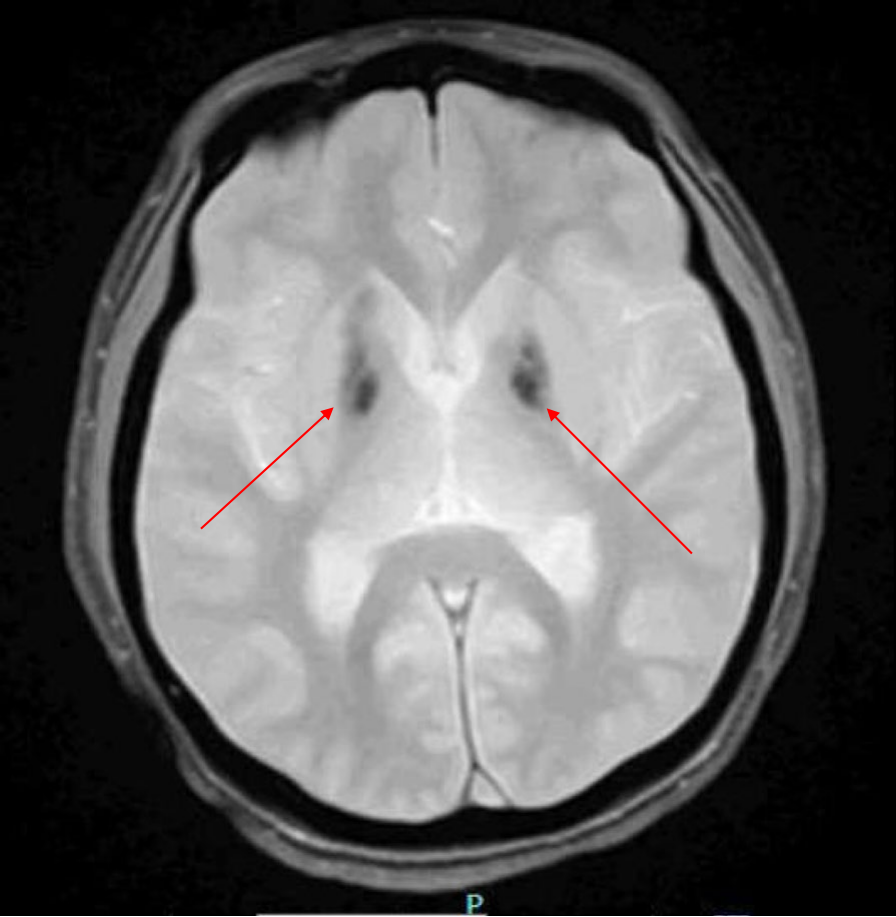
Axial GRE sequence demonstrating symmetric hypodensities in the bilateral deep gray nuclei consistent with calcification

Abnormalities in EEG were found in 26% (*N* = 19/72) of cases. The most frequently reported electrographic feature was epileptiform discharges in the frontal or temporal lobes (58%, *N* = 11/19). Neuroimaging was abnormal in 22% (*N* = 16/72) of cases, with punctate T2 signal abnormalities (81%, *N* = 13/16, Fig. 3) and basal ganglia calcification (18%, *N* = 2/16) (Fig. 4) identified. Two individuals were noted to have incidental structural findings: an anterior temporal arachnoid cyst and Chiari I malformation. Compared to a cohort of individuals with DS who had neuroimaging and met no DSRD exclusion criteria (*n* = 112), only 10 patients (8.9%) had abnormalities on neuroimaging that were not structural (e.g., arachnoid cyst, hypoplastic cerebellum) which was statistically significant compared to individuals with DSRD (*p* < 0.01, 95%CI 1.23–6.85). Nearly all patients in the comparator group were referred to neuroimaging for a comorbid diagnosis of autism spectrum disorder or severe intellectual disability (75%, *N* = 84/112). The remaining individuals were either referred for non-migrainous tension type headache (22%, *N* = 25/112) or rule out of cerebrovascular disease (3%, *N* = 3/112). In this comparator group, abnormalities were punctate T2 signal abnormalities (80%, *N* = 8/10) and calcifications in the basal ganglia (10%, *N* = 1/10) and vermian/midline cerebellum (10%, *N* = 1/10).

Cerebrospinal fluid was abnormal in 17% (9/72) of cases. Pleocytosis was appreciated in five cases (all with > 90% lymphocytosis), elevated total protein in 13% (*N* = 9/72) of cases, elevated IgG index in 12% (*N* = 7/60) of cases, and oligoclonal bands in 3% (*N* = 2/60) of cases. The Mayo Clinic autoimmune encephalopathy panel was negative in all tested cases in both the serum and CSF (*n* = 59). Neopterin was elevated in six cases tested (14%, *N* = 6/43) although pleocytosis and/or elevated total protein was noted in all as well. In cases where the time between diagnosis and neurodiagnostic testing was greater than 3 years, the capture of neurodiagnostic abnormalities was very low (8%, *N* = 2/25) compared to patients receiving assessment prior to 2 years (40%, *N* = 19/47) even though clinical presentations were similar (*p* = 0.01, 95%CI 1.64–37.06).

Clinical symptoms were predictive of some neurodiagnostic study abnormalities (Additional file 4: Appendix 4). Predictors of any neurodiagnostic study abnormality included confusion/disorientation (*p* = 0.01, 95%CI 1.51–34.67), memory impairment (*p* = 0.04, 95%CI 1.07–25.46), catatonia (*p* = 0.04, 95%CI 1.08–16.21), freezing/bradykinesia (*p* = 0.03, 95%CI 1.04–15.63), urinary retention (*p* = 0.02, 95%CI 0.02, 1.20–9.13), and the cognitive/executive (*p* = 0.01, 95%CI 1.28–5.72) and motor symptom clusters (*p* = 0.02, 95%CI 1.09–2.61). Low vitamin D levels were predictive of having any neurodiagnostic abnormalities (*r*^2^ = 0.20, *p* < 0.001, 95%CI 0.01–0.03).

Therapeutic interventions and responses are reported in Table 3. Among all patients, the most effective therapies were IVIg (88%, *N* = 38/43), benzodiazepines (77%, *N* = 46/63), and electroconvulsive therapy (ECT) (74%, *N* = 36/49). Initial immunotherapeutic interventions were either steroids (30 mg/kg/d for 3–5 days, max 1000 mg) or IVIg (2 g/kg divided over 2–3 days followed by monthly infusions of 1g/kg) in all cases. IVIg was markedly effective at improving symptoms with 88% (*N* = 38/43) patients reporting a clinical response as opposed to only 36% (*N* = 14/39) with steroids. The time to the therapeutic effect of steroids or IVIg was rapid at a median of 2.5 weeks (IQR 1–3) in patients who responded. Although less frequently administered, anti-CD20 therapy (750 mg/m^2^, max 1000 mg), mycophenolate (600 mg/m^2^, max 2000 mg/day), and azathioprine (2 mg/kg/day, max 200 mg/day) were also effective in individuals who had both neurodiagnostic abnormalities and a prior response to either steroids or IVIg (Table 3). A review of specific fields of improvement based on therapies utilized is reported in Table 4.

**Table 3:** Therapeutic responses

Therapy type^a^	Utilization (*n* (%))	Effectiveness (*n* (%))			Any neurodiagnostic abnormality vs normal workup		
		All patients (*n* = 72)	Any neurodiagnostic abnormality (*n* = 29)	EEG/MRI/CSF normal (*n* = 43)	*X*^2^ value	*p* value	Odds ratio (95%CI)
Antidepressant	45 (63%)	22 (49%)	4/16 (25%)	18/29 (62%)	5.67	**0.02**	**0.20 (0.05**–**0.79)**
Antipsychotic	52 (72%)	32 (61%)	9/19 (47%)	23/33 (70%)	2.54	0.12	0.39 (0.12–1.26)
Benzodiazepines	63 (87%)	49 (77%)	18/24 (75%)	31/39 (79%)	0.17	0.42	0.77 (0.23–2.59)
ECT	49 (68%)	36 (74%)	6/15 (40%)	30/34 (88%)	12.42	**0.01**	**0.09 (0.02**–**0.39)**
Nutritional therapy	29 (40%)	0 (0%)	0/13 (0%)	0/10 (0%)	0	1.0	n/a
Immunotherapy *Steroids* *IVIg* *Anti-CD20* *MMF/AZ*	43 (59%) 39 (54%) 43 (59%) 19 (26%) 19 (26%)	74/120 (62%) 14/39 (36%) 38/43 (88%) 9/19 (47%) 13/19 (68%)	55/74 (74%) 10/24 (42%) 24/26 (92%) 9/11 (81%) 12/13 (92%)	19/46 (41%) 4/15 (27%) 14/17 (82%) 0/8 (0%) 1/6 (17%)	10.04 0.90 0.05 9.89 12.17	**< 0.001** 0.34 0.33 **0.01** **0.01**	**4.11 (1.88**–**9.02)** 1.96 (0.48–7.99) 2.57 (0.38–17.31) **49.5 (3.84**–**638.43)** **60.0 (3.10**–**1159.84)**

*AZ* Azathioprine, *CSF* cerebrospinal fluid, *EEG* electroencephalogram, *ECT* electroconvulsive therapy, *MRI* magnetic resonance imaging, *MMF* mycophenolate mofetil

^a^Patients may have received multiple therapeutic interventions creating a higher “*n*” with regard to the treatment interventions by class

**Table 4:** Type of therapeutic response in individuals who responded to therapy

Therapy type	All patients (*n* = 72)				Neurodiagnostic study abnormality (*n* = 29)				EEG/MRI/CSF normal (*n* = 43)			
	*SI%*	*RL%*	*DC%*	*PE%*	*SI%*	*RL%*	*DC%*	*PE%*	*SI%*	*RL%*	*DC%*	*PE%*
**Antidepressant** (*n* = 45)	46	–	29 (13/45)	40 (18/45)	34	0 (0/16)	12 (2/16)	25 (4/16)	54	–	38 (11/29)	48 (14/29)
**Antipsychotic** (*n* = 52)	41	–	33 (17/52)	35 (18/52)	28	0 (0/19)	5 (1/19)	21 (4/19)	49	–	48 (16/33)	43 (14/33)
**Benzodiazepine** (*n* = 63)	57	–	63 (40/63)	87 (55/63)	41	0 (0/24)	52 (13/25)	72 (18/25)	68	–	70 (27/39)	95 (37/39)
**ECT** (*n* = 49)	65	–	24 (12/49)	59 (29/49)	32	0 (0/15)	13 (2/15)	40 (6/15)	87	–	29 (10/34)	68 (23/34)
**Nutritional therapy** (*n* = 29)	0	–	0 (0/29)	0 (0/29)	0	0 (0/13)	0 (0/13)	0 (0/13)	0	–	0 (0/10)	0 (0/10)
**Immunotherapy** (*n* = 43) *Steroids* *IVIg* *Anti-CD20* *MMF/AZ*	68 27 73 33 38	– – – – –	48 (58/120) 5 (2/39) 77 (33/43) 58 (11/19) 63 (12/19)	59 (71/120) 26 (10/39) 88 (38/43) 53 (10/19) 68 (13/19)	72 36 82 63 75	47 (9/19) 0 (0/4) 50 (4/8) 67 (2/3) 75 (3/4)	58 (43/74) 8 (2/24) 73 (19/26) 100 (11/11) 85 (11/13)	73 (54/74) 30 (7/24) 96 (25/26) 91 (10/11) 92 (12/13)	45 20 68 0 20	– – – – –	33 (15/46) 0 (0/15) 82 (14/17) 0 (0/8) 17 (1/6)	37 (17/46) 20 (3/15) 76 (13/17) 0 (0/8) 17 (1/6)

*Note*: Individuals without neurodiagnostic study abnormalities could not display improvement, and thus, only individuals with these findings are reported. Not all patients with neurodiagnostic abnormalities had repeat testing

*AZ* Azathioprine, *CSF* cerebrospinal fluid, *EEG* electroencephalogram, *ECT* electroconvulsive therapy, *DC* discontinuation of medication, *MRI* magnetic resonance imaging, *MMF* mycophenolate mofetil, *PE* physical or neurologic examination improvement, *RL* resolution of lab or neurodiagnostic abnormality, *SI%* subjective improvement percentage

In individuals with neurodiagnostic abnormalities, the use of immunotherapy was nearly four times more likely to have a therapeutic effect compared to individuals without neurodiagnostic abnormalities (OR 4.11, *p* = 0.001, 95%CI 1.88–9.02). In those with normal neurodiagnostic studies, IVIg was effective in 82% (*N* = 14/17) who received it empirically although only one other patient had clinical improvement with other forms of non-steroid immunotherapy (7%, *N* = 1/14). The effectiveness of antipsychotics (*p* = 0.12, 95%CI 0.12–1.26) and benzodiazepines (*p* = 0.42, 95%CI 0.23–2.59) was not significantly different between the groups. Antidepressants and ECT were more likely to be effective in individuals without neurodiagnostic abnormalities compared to those with these findings (OR 0.20, *p* = 0.02, 95%CI 0.05–0.79 and OR 0.09, *p* = 0.04, 95%CI 0.02–0.39, respectively).

## Discussion

This study reports the presence of multiple neurodiagnostic study abnormalities in nearly half of individuals with DSRD and preferential response to immunotherapy in individuals with these confirmed abnormalities. These findings support the possibility that, in a minority of individuals with DSRD, neuroimmunologic and neuroinflammatory etiologies can potentially yield the phenotypic symptom cluster described in the literature. Of note, the rate of neurodiagnostic abnormality capture was markedly higher in individuals receiving diagnostic assessment within 2 years of symptom onset, highlighting the importance of prompt and aggressive neurodiagnostic workup for this symptom cluster.

The significance of neurodiagnostic study abnormalities in persons with DSRD cannot be overstated. Limited case reports in individuals with DSRD have identified CSF anomalies although the variability in these cases made interpretation challenging [15]. In our cohort, 17% of individuals had some form of CSF abnormality. Although there are no existing normative values for individuals with DS, the presence of CSF pleocytosis [16], elevated CSF protein [16, 17], presence of restricted oligoclonal bands [18, 19], elevated IgG index [16, 19], and elevated neopterin [20–22] have all been linked to the presence of neuroinflammatory disorders in children. The presence of these abnormalities, and the overlap between them and other neurodiagnostic studies, provides preliminary evidence for central nervous system dysfunction in individuals with DSRD. Individuals with DS are predisposed to polyfactorial immune dysregulation which can include interferon signaling [23–25], T-cell function [26–28], and B-cell/antibody-mediated disease [29, 30], making the determination of the defective pathway in DSRD challenging. Further research is needed to differentiate the causative pathways of disease as the lab-based CSF abnormalities indicate a combination of both T-cell (elevated neopterin and total protein) and B-cell (restricted oligoclonal bands and elevated IgG index) disease in individuals with DSRD.

Although neurodiagnostic anomalies were observed, no specific pattern emerged as predictive of the disease or response, yielding the need to interpret all studies as biomarkers of cerebral dysfunction as opposed to being diagnostic or confirmatory of DSRD. Although serum data was of limited value due to low *n* in this study, it is noteworthy that elevations in IL-10 were observed as chromosome 21 encodes for the beta subunit of this interleukin and may provide a fast-forward mechanism of disease in DSRD given that levels of this interleukin have previously been identified as no different than neurotypical individuals [31]. Elevations in IL-10 were present in both individuals with DS alone and DSRD, which is confirmatory of this hypothesis, although there was a statistically significant difference in the presence of elevations in sIL2 and TNF-alpha in individuals with DSRD compared to DS alone indicating that the former cohort has a potential cytokine signaling and/or inflammatory pathology present.

In our cohort, individuals with DSRD and neurodiagnostic study abnormalities experience a nearly fourfold greater likelihood of response to immunotherapy compared to those without. This observation is particularly striking in that the observed therapeutic response validates the clinical significance of the neurodiagnostic abnormalities observed. The therapeutic response reported builds on prior reports of immunotherapy-responsive DSRD in individuals with neurodiagnostic study abnormalties [15, 32]. Although steroids are well established as first-line therapy in a variety of neuro-immunologic disorders [33–37], efficacy was lower with regard to clinical improvement. The reason for this remains unclear although could potentially be related to heterogeneity in administration (oral versus intravenous) and use in a population likely to experience side effects due to medical comorbidities which may obscure clinical improvement. Among individuals who responded to IVIg, the use of other immunotherapeutics was nearly uniformly beneficial in individuals with or without neurodiagnostic study abnormalities. This highlights the possibility that while IVIg may be beneficial for the treatment of DSRD, primary neuroinflammation is not likely present in all cases, requiring a more nuanced assessment of the need for second-line immunotherapy by physicians. This has been previously observed in rare observational cohorts of individuals with rare genetic disorders that predispose toward immunotherapy-responsive neuroinflammation such as SHANK3 and Aicardi-Goutières syndrome [14, 38]. Another observation from our therapeutic response data is that the clinical improvements associated with first-line immunotherapy (steroids and IVIg) were clear although non-specific given that individuals without neurodiagnostic abnormalities also responded to these treatments. Second-line therapeutics such as mycophenolate, azathioprine, and methotrexate, when used, were highly effective although only in individuals with neurodiagnostic, and specifically CSF-based, abnormalities. Although the interpretation of this data is limited by a small number of patients, it is confirmatory that traditional immunotherapy is of great value in individuals with DSRD and evidence of neuroinflammation.

Individuals without neurodiagnostic abnormalities were twice as likely to respond to antipsychotics, five times more likely to respond to anti-depressants, and ten times more likely to respond to ECT, the latter two being statistically significant differences. Strong data exists for the higher prevalence of a variety of psychiatric disorders in persons with DS [39], and thus, successful treatment of these psychiatric comorbidities may be reflective of psychiatric disease as the etiology of DSRD in these patients [40, 41]. That being said, the authors acknowledge that the link between stress, psychiatric disease, neurologic disease, and the immune system remains unknown although may prove to be a point of interface in shared etiologies of DSRD [42, 43]. Ultimately, DSRD is a symptom cluster, and thus, multiple etiologies for this condition are highly likely. These findings provide preliminary evidence that distinct etiologies of DSRD may respond differently to therapeutic interventions, highlighting the need for thorough clinical assessment and neurodiagnostic study obtainment. Further supporting this concept is the identification that all individuals with DSRD onset < 8 years were not responsive to immunotherapy.

Assessing CNS inflammation and autoimmunity poses many challenges, including current testing modalities that are insufficient to capture the breadth of immune-mediated processes. Ongoing discoveries in the area of autoimmune encephalitis (AE) have highlighted these limitations and revealed that a substantial number of cases may have unremarkable neurodiagnostic studies during their disease course, regardless of disease activity [44–46]. While the overall response to immunotherapy was lower in individuals without neurodiagnostic abnormalities in our cohort, it continues to remain possible that capture of neurodiagnostic abnormalities may be time-dependent, which was appreciated in the lower yield of testing abnormalities in individuals with a longer time between symptom onset and diagnosis. Thus, it could be argued that the obtainment of neurodiagnostic studies could be more beneficial for guiding second-line immunotherapy than for the initiation of primary immunotherapeutics such as steroids and IVIg, which carry a low side effect profile and marked efficacy in both sub-groups. However, the data builds upon prior reports [15, 32] of the use of immunotherapy in individuals with DSRD regardless of neurodiagnostic study results. While the therapeutic response in both sub-populations is exciting, the interpretation of this data must be tempered by the non-randomized, non-controlled nature of the study.

A question that emerges from this study is whether DSRD is a form of AE given the temporal and symptomatic overlap between the conditions [33, 47]. The lack of definitive autoantibody capture and a phenotype unlike any established form of AE that has been identified argues for a distinct entity [48]. In addition, younger patients have a much higher rate of seizures in the setting of autoimmune encephalitis which was not reported in this cohort [16, 33]. Finally, when utilizing Graus et al.’s criteria [47], only four patients (6%) met the criteria for autoantibody-negative but probable AE. It is the opinion of the authors that in a minority of individuals with DSRD, there may be similar neuroinflammatory mechanisms at play as in AE although, ultimately, this process is likely unique.

This study is not without limitations. Firstly, the retrospective nature of this study introduces selection bias and recall bias. While the authors attempted to mitigate this by having very strict inclusion/exclusion criteria, this also reduced the total “*n*” for this study, which already reports a rare disease entity. Most individuals were evaluated in neuroimmunology clinics, introducing the potential for ascertainment and confirmation. This study required a substantive subjective review of data for analysis although this was controlled using a two-tiered review system with a neutral arbiter. The subjective nature of the report and the lack of specific grading systems or diagnostic criteria for DSRD did make this challenging. For this reason, a large functional decline (> 50%) was utilized as an inclusion criterion, ensuring that more definitive cases were included but more marginal or questionable cases would not be. This may have excluded individuals with mild or moderate DSRD phenotypes from the study, creating the potential for a severity bias. In the absence of clear guidelines, this was felt to be the most conservative method of ensuring the fidelity of patients reviewed. Our study’s high Cohen’s kappa (0.72) indicated substantial agreement between reviewers, lowering the risk of misinterpretation of data by single reviewers. Although data extraction was standardized, it ultimately relied on parental, caregiver, or physician report, which is subjective and susceptible to bias. Similarly, efficacy was interpreted broadly, and particular therapies may have resulted in isolated symptomatic improvement of symptoms as opposed to the underlying disease process (e.g., benzodiazepines improving catatonia but not DSRD). Another confounder is that the median time from onset to evaluation in this study was 2 years. This may have caused a higher likelihood to detect some neurodiagnostic abnormalities (EEG/MRI) and a lower likelihood to detect others (CSF). Furthermore, the report of neurodiagnostic study abnormalities is challenging in that no true control values are available for this cohort. Children with DS do not routinely receive EEG, neuroimaging, or lumbar puncture nor is there “control” data to compare to. For instance, children with DS will only typically receive a study like a lumbar puncture if there is a clinical concern (e.g., meningitis) which makes comparison against a “typical” child with DS very challenging. This limited our neurodiagnostic comparisons in this study to only neuroimaging. Similarly, certain aspects of clinical information in “control” patients, such as exposure to potential triggers, could not be collected due to the retrospective nature of the study and that this data is not typically collected at the standard of care visits. This highlights the importance of a controlled trial being the logical next step to expand on the data presented in this manuscript. The restrictive inclusion/exclusion criteria of this study may have also elevated the rate of capture for neurodiagnostic abnormalities by way of excluding patients with a lower likelihood neurologic disease. A critical limitation in this study is that the data presented is retrospective, non-randomized, and non-controlled, which must augment the interpretation of therapeutic responses. As such, the authors elected to not perform statistical analysis on different types of therapeutic responses reported as these were felt to be subjective and could lead to misinterpretation or overinterpretation of the data causing type I error. While exciting, prospective data must be collected before uniform utilization of these interventions is made. Finally, workups were heterogeneous in this cohort which limits the power and generalizability of some of the findings reported, especially in that there are no well-studied reference ranges for individuals with DS without DSRD for many of these labs.

### Conclusions

This study reports the novel presence of electrographic, neuroimaging, and CSF testing abnormalities in individuals with DSRD, providing credence to this symptom cluster being neurologic and/or neuroimmunologic in origin in a minority of cases. Given the importance of identifying a potential etiology of this disease and the marked therapeutic effect of immunotherapy in persons with neurodiagnostic abnormalities, further study into the role of neuroinflammation in DSRD is warranted and desperately needed.

## Supplementary Information

**Additional file 1: Appendix 1.** List of contributing sites.

**Additional file 2: Appendix 2.** Symptom clusters for individuals with DSRD.

**Additional file 3: Appendix 3.** Definitions of “abnormal” on neurodiagnostic studies.

**Additional file 4: Appendix 4.** Clinical symptoms and prediction of neurodiagnostic abnormalities.

## Data Availability

Anonymized data will be made available to qualified investigators upon request and IRB approval for release.

## Abbreviations

AE: Autoimmune encephalitis
CSF: Cerebrospinal fluid
DS: Down syndrome
DSDD: Down syndrome disintegrative disorder
DSRD: Down syndrome regression disorder
ECT: Electroconvulsive therapy
EEG: Electroencephalogram
LP: Lumbar puncture
IVIg: Intravenous immunoglobulin
MAR: Medication administration record
MRI: Magnetic resonance imaging
OSA: Obstructive sleep apnea
URDS: Unexplained regression in Down syndrome
