# Supplementary material for: Evidence of neuroinflammation and immunotherapy responsiveness in individuals with down syndrome regression disorder
Source: J Neurodev Disord. 2022 Jun 3;14:35. doi: 10.1186/s11689-022-09446-w (PMC9164321; doi:10.1186/s11689-022-09446-w)
Supplement: Supplementary file 1 — Additional file 1: Appendix 1. List of contributing sites. [file 11689_2022_9446_MOESM1_ESM.docx]

**Appendix 1:** List of contributing sites

Down Syndrome Program at Virginia Mason, Seattle, WA

University of Colorado Medical Center/Denver Health, Aurora, CO

Children’s Healthcare of Atlanta and Emory University School of Medicine, Atlanta, GA

Cincinnati Children’s Hospital, Cincinnati, OH

Ochsner Health, New Orleans, LA

Tulane University Medical Center, New Orleans, LA

Down Syndrome Program, Stanford University, Palo Alto, CA

Down Syndrome Program, Massachusetts General Hospital, Boston, MA

University of Toledo School of Medicine, Toledo, OH

Children’s Hospital Los Angeles, Los Angeles, CA
