# Supplementary material for: Evidence of neuroinflammation and immunotherapy responsiveness in individuals with down syndrome regression disorder
Source: J Neurodev Disord. 2022 Jun 3;14:35. doi: 10.1186/s11689-022-09446-w (PMC9164321; doi:10.1186/s11689-022-09446-w)
Supplement: Supplementary file 2 — Additional file 2: Appendix 2. Symptom clusters for individuals with DSRD. [file 11689_2022_9446_MOESM2_ESM.docx]

**Appendix 2:** Symptom clusters for individuals with DSRD

1. Mood (social withdrawal, loss of acquired skills, mutism, and whispered speech)
2. Cognitive/executive function (impaired attention, reduced eye contact, confusion/disorganization, memory impairment)
3. Motor (abulia/avolition, stereotypy, tics, catatonia, freezing or bradykinesia)
4. Behavioral (apathy/withdrawal, hyperactivity, inappropriate laughter, aggression and agitation)
5. Sleep (insomnia and circadian rhythm disruption)
6. Bowel/bladder (incontinence and urinary retention)
7. Neurologic (autonomic dysfunction, seizure(s), focal neurologic deficits, and transient ischemic attack)
8. Psychiatric (anxiety, obsessive compulsive disorder, anorexia, and emotional lability)
