# Supplementary material for: Evidence of neuroinflammation and immunotherapy responsiveness in individuals with down syndrome regression disorder
Source: J Neurodev Disord. 2022 Jun 3;14:35. doi: 10.1186/s11689-022-09446-w (PMC9164321; doi:10.1186/s11689-022-09446-w)
Supplement: Supplementary file 3 — Additional file 3: Appendix 3. Definitions of “abnormal” on neurodiagnostic studies. [file 11689_2022_9446_MOESM3_ESM.docx]

**Appendix 3:** Definitions of “abnormal” on neurodiagnostic studies

1. Electroencephalogram (EEG): Focal or generalized slowing, focal epileptiform discharges out of any cortex, or seizure were considered abnormal. Generalized discharges were considered abnormal although inconsistent with the diagnosis of DSRD. All patients had to have at least one prior EEG that did not demonstrate these results previously.
2. MRI: All MRIs had to be performed on a 3T scanner with and without contrast administration. Any abnormality beyond a structural malformation (e.g., Chiari malformation) was considered abnormal. Patients did not require a prior “normal” MRI.
3. Lumbar Puncture (LP): Abnormalities were defined as having any of the following: WBC count >5 cells/mm3, total protein >60 mg/dL, presence of oligoclonal bands, an IgG index of >0.66, and/or an elevated neopterin (>33 nmol/mL). Samples with over 1,000 RBC were excluded from analysis. Patients did not require a prior “normal” LP.
