# Supplementary material for: Evidence of neuroinflammation and immunotherapy responsiveness in individuals with down syndrome regression disorder
Source: J Neurodev Disord. 2022 Jun 3;14:35. doi: 10.1186/s11689-022-09446-w (PMC9164321; doi:10.1186/s11689-022-09446-w)
Supplement: Supplementary file 4 — Additional file 4: Appendix 4. Clinical symptoms and prediction of neurodiagnostic abnormalities. [file 11689_2022_9446_MOESM4_ESM.docx]

**Appendix 4:** Clinical symptoms and prediction of neurodiagnostic abnormalities·

| **Symptom Cluster** | **EEG**  (OR, *p* value, 95%CI) | **MRI**  (OR, *p* value, 95%CI) | **LP**  (OR, *p* value, 95%CI) | **Any Neurodiagnostic**  (OR, *p* value, 95%CI) |
| --- | --- | --- | --- | --- |
| **Mood**  *Social withdrawal*  *Loss of Acquired Skills*  *Mutism*  *Whispered speech* | 1.22 (0.67, 0.49-3.03)  -  -  0.93 (0.91, 0.24-3.51)  1.72 (0.75, 0.42-7.08) | 1.55 (0.40, 0.56-4.33)  -  -  1.85 (0.46, 0.35-9.68)  1.49 (0.59, 0.36-6.23) | 1.79 (0.36, 0.51-6.35)  -  -  1.19 (0.84, 0.22-6.52)  3.34 (0.26, 0.39-30.01) | 1.56 (0.29 (0.69-3.57)  -  -  1.65 (0.44, 0.46-5.99)  1.65 (0.41, 0.51-5.38) |
| **Cognitive/Executive Function**  *Impaired attention*  *Reduced eye contact*  *Confusion/disorganization*  *Memory impairment* | **2.37 (0.05, 1.01-5.63)**  0.66 (0.59, 0.14-3.10)  1.35 (0.80, 0.13-13.89)  **9.12 (0.04, 1.11-75.27)**  1.94 (0.36, 0.47-7.91) | **3.83 (0.01, 1.30-11.26)**  1.97 (0.55, 0.21-18.33)  1.2 (0.88, 0.12-12.41)  3.75 (0.11, 0.75-18.74)  5.81 (0.11, 0.69-48.92) | **10.99 (0.02, 1.46-85.57)**  1.45 (0.75, 0.15-13.73)  0.83 (0.87, 0.08-8.73)  **8.69 (0.05, 1.02-73.99)**  **10.45 (0.03, 1.23-88.60)** | **2.71 (0.01, 1.28-5.72)**  1.14 (0.87, 0.25-5.19)  2.18 (0.51, 0.22-21.98)  **7.23 (0.01, 1.51-34.67)**  **5.23 (0.04, 1.07-25.46)** |
| **Motor**  *Abulia/avolition*  *Stereotypy*  *Tics*  *Catatonia*  *Freezing/bradykinesia* | 1.25 (0.38, 0.76-2.07)  1.07 (0.91, 0.34-3.31)  0.95 (0.93, 0.32-2.87)  0.74 (0.66, 0.20-2.75)  3.86 (0.09, 0.78-19.18)  1.69 (0.42, 0.47-6.08) | 1.35 (0.26, 0.79-2.29)  1.50 (0.52, 0.44-5.09)  0.95 (0.94, 0.30-3.01)  0.97 (0.97, 0.16-5.92)  3.38 (0.14, 0.67-16.96)  2.09 (0.30, 0.51-8.55) | 1.13 (0.69, 0.62-2.06)  1.81 (0.43, 0.42-7.83)  0.55 (0.38, 0.14-2.13)  0.91 (0.89, 0.21-4.01)  1.29 (0.74, 0.29-5.61)  2.17 (0.36, 0.41-11.42) | **1.69 (0.02, 1.09-2.61)**  1.76 (0.27, 0.64-4.96)  1.17 (0.74, 0.46-3.02)  0.81 (0.69, 0.27-2.39)  **4.18 (0.04, 1.08-16.21)**  **4.02 (0.04, 1.04-15.63)** |
| **Behavioral**  *Apathy/withdrawal*  *Hyperactivity*  *Inappropriate laughter*  *Aggression/agitation* | 0.99 (0.99, 0.57-1.75)  1.95 (0.56, 0.20-18.69)  0.78 (0.76, 0.14-4.24)  0.67 (0.49, 0.21-2.10)  0.96 (0.95, 0.28-3.29) | 0.94 (0.84, 0.52-1.69)  1.73 (0.64, 0.18-16.72)  1.42 (0.65, 0.31-6.53)  0.95 (0.93, 0.28-3.22)  0.16 (0.09, 0.02-1.39) | 1.13 (0.73, 0.57-2.24)  1.19 (0.88, 0.12-11.77)  3.52 (0.10, 0.79-15.81)  0.76 (0.71, 0.18-3.26)  0.26 (0.22, 0.03-2.34) | 1.1 (0.69, 0.68-1.78)  3.14 (0.32, 0.33-29.59)  1.28 (0.70, 0.35-4.68)  1.11 (0.84, 0.41-2.99)  0.63 (0.41, 0.21-1.92) |
| **Sleep**  *Insomnia*  *Circadian rhythm alteration* | 0.99 (0.98, 0.45-2.17)  0.59 (0.43, 0.16-2.15)  1.47 (0.51, 0.46-4.63) | 1.32 (0.51, 0.57-3.03)  0.69 (0.59, 0.17-2.69)  2.31 (0.16, 0.72-7.48) | 1.14 (0.78, 0.44-8.96)  0.61 (0.53, 0.13-2.82)  1.92 (0.34, 0.49-7.44) | 1.29 (0.46, 0.66-2.56)  0.88 (0.83, 0.27-2.86)  1.88 (0.21, 0.70-4.99) |
| **Bowel/Bladder**  *Incontinence*  *Urinary retention* | 1.29 (0.53, 0.59-2.80)  0.67 (0.48, 0.22-2.04)  2.53 (0.11, 0.79-7.98) | 1.92 (0.13, 0.83-4.40)  1.35 (0.61, 0.42-4.27)  2.78 (0.09, 0.82-9.38) | 2.01 (0.16, 0.77-5.29)  1.26 (0.74, 0.33-4.75)  3.37 (0.10, 0.78-14.46) | 1.63 (0.16, 0.83-3.19)  1.12 (0.81, 0.44-2.88)  **3.32 (0.02, 1.2-9.13)** |
| **Neurologic**  *Autonomic Dysfunction*  *Seizure*  *Focal neurologic deficit*  *Transient ischemic attack* | **3.63 (0.03, 1.11-11.86)**  1.86 (0.45, 0.36-9.66)  2.69 (0.49, 0.16-45.57)  5.25 (0.19, 0.44-61.98)  2.53 (0.52, 0.15-42.78) | **3.51 (0.4, 1.03-11.97)**  1.5 (0.67, 0.24-9.46)  2.87 (0.47, 0.17-48.75)  6.0 (0.16, 0.50-71.33)  2.87 (0.47, 0.17-48.75) | **4.16 (0.03, 1.15-15.09)**  1.88 (0.51, 0.29-12.14)  4.3 (0.32, 0.25-74.78)  9.33 (0.08, 0.76-114.40)  - | 2.48 (0.12, 0.79-7.79)  1.50 (0.59, 0.34-6.70)  1.59 (0.75, 0.09-26.54)  3.11 (0.36, 0.27-36.01)  1.54 (0.77, 0.09-25.57) |
| **Psychiatric**  *Anxiety*  *OCD*  *New autistic features*  *Anorexia*  *Emotional lability* | 0.97 (0.92, 0.57-1.67)  3.31 (0.28, 0.38-29.05)  0.59 (0.42, 0.16-2.13)  -  1.94 (0.36, 0.47-7.91)  0.54 (0.32, 0.16-1.81) | 1.44 (0.24, 0.78-2.63)  2.92 (0.34, 0.33-25.81)  1.24 (0.72, 0.38-4.11)  -  2.71 (0.23, 0.53-13.76)  1.26 (0.73, 0.35-4.57) | 1.36 (0.39, 0.68-2.71)  1.94 (0.56, 0.21-17.71)  1.18 (0.81, 0.29-4.72)  -  4.26 (0.19, 0.49-36.66)  0.70 (0.62, 0.17-2.88) | 1.28 (0.31, 0.79-2.07)  1.69 (0.48, 0.39-7.14)  1.09 (0.87, 0.40-2.95)  -  **3.76 (0.05, 1.02-14.64)**  1.19 (0.74, 0.42-3.36) |

*Legend:* electroencephalogram (EEG), lumbar puncture (LP), magnetic resonance imaging (MRI), obsessive compulsive disorder (OCD), odds ratio (OR).
